# Effect of Low Dose Docosahexaenoic Acid-Rich Fish Oil on Plasma Lipids and Lipoproteins in Pre-Menopausal Women: A Dose–Response Randomized Placebo-Controlled Trial

**DOI:** 10.3390/nu10101460

**Published:** 2018-10-08

**Authors:** Cassandra Sparkes, Robert Gibson, Andrew Sinclair, Paul L. Else, Barbara J. Meyer

**Affiliations:** 1 School of Medicine, University of Wollongong, Wollongong, NSW 2522, Australia; sparkesc@gmail.com (C.S.); pelse@uow.edu.au (P.L.E.); 2 Illawarra Medical Research Institute, University of Wollongong, Wollongong, NSW 2522, Australia; 3 University of Adelaide, Adelaide, SA 5005, Australia; robert.gibson@adelaide.edu.au; 4 Faculty of Health, Deakin University, Geelong, VIC 3220, Australia; Andrew.sinclair@deakin.edu.au; 5 Department of Nutrition, Dietetics and Food, Monash University, Notting Hill, VIC 3168, Australia

**Keywords:** DHA, plasma lipids, lipoproteins, premenopausal women

## Abstract

Omega-3 long chain polyunsaturated fatty acid (*n*-3 LCPUFA) supplementation has been shown to improve plasma lipid profiles in men and post-menopausal women, however, data for pre-menopausal women are lacking. The benefits of intakes less than 1 g/day have not been well studied, and dose–response data is limited. The aim of this study was to determine the effect of low doses of docosahexaenoic acid (DHA)-rich tuna oil on plasma triglyceride (TG) lowering in pre-menopausal women, and investigate if low dose DHA-rich tuna oil supplementation would increase the low-density lipoprotein (LDL) and high-density lipoprotein (HDL) particle sizes. A randomized, double-blind, placebo-controlled trial was conducted, in which 53 healthy pre-menopausal women with mildly elevated plasma TG levels consumed 0, 0.35, 0.7, or 1 g/day *n*-3 LCPUFA as HiDHA™ tuna oil or placebo (Sunola oil) capsules for 8 weeks. Supplementation with 1 g/day *n*-3 LCPUFA, but not lower doses, reduced plasma TG by 23% in pre-menopausal women. This was reflected in a dose-dependent reduction in very-low-density lipoprotein (VLDL)-TG (*R*^2^ = 0.20, *p* = 0.003). A weak dose-dependent shift in HDL (but not LDL) particle size was identified (*R*^2^ = 0.05, *p* = 0.04). The results of this study indicate that DHA-rich *n*-3 LCPUFA supplementation at a dose of 1 g/day is an effective TG-lowering agent and increases HDL particle size in pre-menopausal women.

## 1. Introduction

The ability of omega-3 long chain polyunsaturated fatty acid (*n*-3 LCPUFA) to reduce plasma triglycerides is well established [1,2], and high dose prescriptions of *n*-3 LCPUFA are commercially available for treatment of hypertriglyceridemia [3]. High dose *n*-3 LCPUFA supplementation may concurrently raise low-density lipoprotein-cholesterol (LDL-C) levels; however, this may be counteracted by a shift in LDL particle size towards the larger, less atherogenic LDL_1_ particles [4]. Eicosapentaenoic acid (EPA) and docosahexaenoic acid (DHA) appear to lower plasma triglyceride (TG) to a similar extent [5], however, DHA seems to be more effective in producing beneficial changes to LDL and high-density lipoprotein (HDL) particle sizes [5,6,7,8]. Yet, while the benefits of higher doses (>3 g/day) of *n*-3 LCPUFA on plasma lipids and lipoproteins are well-established, much less is known about the benefits of lower doses (<1 g/day). It is important to understand the benefits at these intake levels, given that they are in the range of recommended dietary intakes.

A dose-dependent lowering of plasma TG (*R*^2^ = 0.29) was reported in men and women at moderate intakes (0–2 g/day) [9], however, the current understanding of dose–response relationships at lower intakes is limited. Also, the majority of studies have been conducted in men [1] and post-menopausal women [10,11,12], who inherently have less favourable HDL-C and HDL_2_ levels than pre-menopausal women [13]. Placebo-controlled trials have not been conducted exclusively in pre-menopausal women to investigate the TG-lowering potential of *n*-3 LCPUFA. Studies have been conducted in mixed gender populations [12,14,15,16], however, the majority have failed to report on sex differences in lipid responses. Furthermore, sex hormone fluctuations alter blood lipids and lipoproteins [17,18], and the potentially confounding effects of plasma lipid changes throughout the menstrual cycle have rarely been taken into account. Research on the TG-lowering effects of *n*-3 LCPUFA is especially important in women, given that plasma TGs are a stronger determinant of coronary heart disease (CHD) risk in women than men [19,20]. Also, while the majority of studies have found no effect of low doses of *n*-3 LCPUFA on blood pressure [21], reductions in systolic [22] and diastolic [16] blood pressure have been observed with just 0.7–1 g/day of DHA or a DHA-rich supplement.

The primary aim of this study was to determine a low dose–response effect of DHA-rich tuna oil supplementation for plasma TG lowering in pre-menopausal women. Secondary aims were to determine if the supplementation reduces very-low-density lipoprotein (VLDL)-TG and intermediate-density lipoprotein (IDL)-TG, increases the larger HDL (HDL_2_) particles, and increases LDL particle size from small dense LDL_3_ to normal-sized LDL particles, in a dose–response manner, as well as determining whether it lowers blood pressure.

## 2. Materials and Methods

### 2.1. Subjects

**Figure 1:**
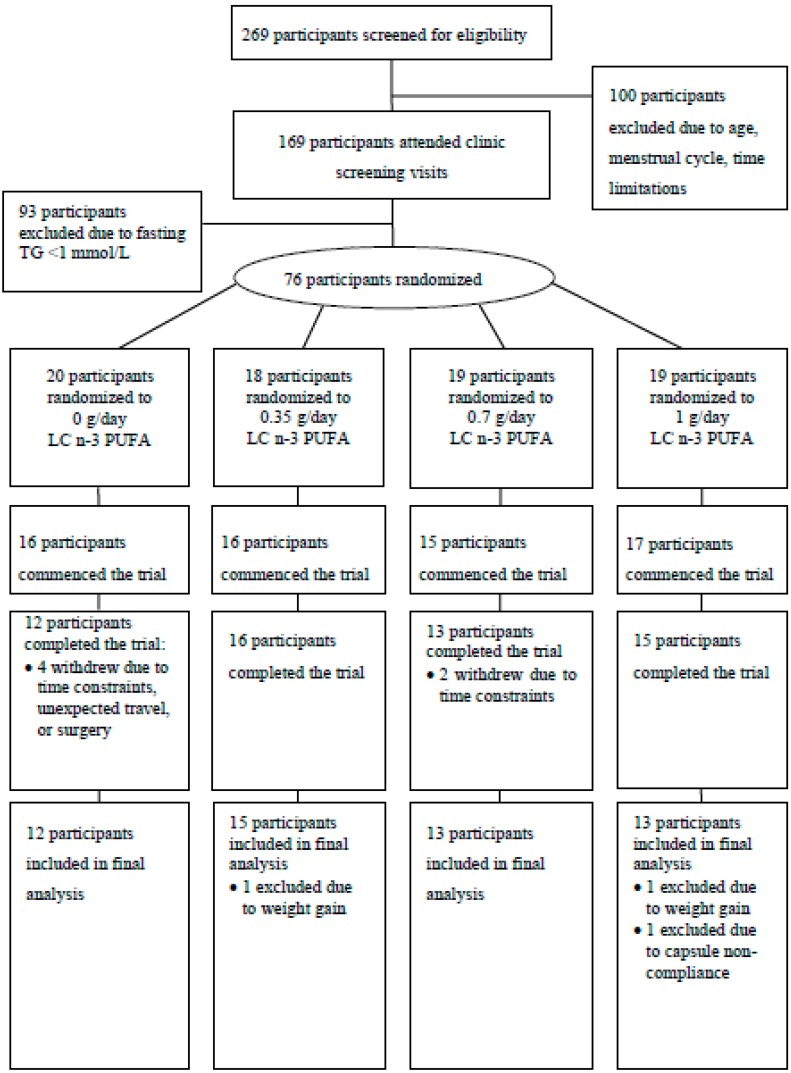
Flow of participants through the double-blind randomized placebo-controlled trial. TG: triglyceride; *n*-3 LCPUFA: omega-3 long chain polyunsaturated fatty acid.

Healthy premenopausal women with mildly elevated triglycerides (>1.0 mmol/L) and regular menstrual cycles (28–32 days) were recruited for this study conducted at the University of Wollongong, Australia. Exclusion criteria included age <18 or >40 years, consumption of fish oil supplements, and known existing cardiovascular disease (CVD). Subjects who completed the trial were required to achieve a capsule compliance rate of >90% on the basis of self-report and excess capsule count, and a body weight change of ≤5% from baseline. Subjects were recruited through advertisement in the local media (television, radio, newspaper), distribution of fliers in the community, and University email lists. Of the 269 women who participated in phone screening, 169 were eligible to attend screening clinic visits (Figure 1). These subjects attended the research clinic for measurement of fasting plasma TG levels, and their height and weight were recorded for calculation of body mass index (BMI). Upon exclusion of 93 subjects due to low plasma TGs (<1.0 mmol/L), 76 women were eligible to participate in the placebo-controlled trial. The study protocol was approved by the Human Research Ethics Committee of the University of Wollongong, and written informed consent was obtained from subjects (Australian and New Zealand Clinical Trial Registration ID: ANZCTRN12607000566437).

### 2.2. Study Design

A randomized, double-blind, placebo-controlled trial of parallel design was conducted, whereby subjects were randomly assigned to consume 0, 0.35, 0.7, or 1.0 g/day of *n*-3 LCPUFA for two menstrual cycles (approximately 8 weeks). Randomization was controlled for age, BMI, and contraceptive pill use. *n*-3 LCPUFA was provided in the form of HiDHA™ tuna oil (500 mg sized capsules supplied by Nu-Mega Ingredients, Australia). Subjects consumed six capsules daily, with the four different doses achieved by varying the proportion of active (HiDHA™ tuna oil) and placebo (Sunola oil, 500 mg) capsules for each dose group. Each tuna oil capsule provided 135 mg DHA and 35 mg EPA, whereas each Sunola capsule provided 355 mg monounsaturated fatty acids (MUFA), 55 mg saturated fatty acids (SFA), and 14 mg polyunsaturated fatty acids (PUFA) (no *n*-3 LCPUFA). Daily doses of capsules were provided in individual zip-lock bags, and an excess of these bags were distributed to subjects. Subjects also kept a diary to record daily capsule intake and menstrual cycle status. Compliance with capsule consumption was assessed using the diary records and excess capsule count, and confirmed by measurement of erythrocyte *n*-3 LCPUFA levels. Subjects attended the research clinic on two consecutive mornings each at baseline, and after approximately 8 weeks (two menstrual cycles) of supplementation. In order to control the confounding effects of the menstrual cycle, the clinic visits were conducted on days 3–5 of the menstrual cycle, as determined by counting from onset of menses, and thus were strictly aligned with the onset of menses. This alignment discounts the effect of the hormonal influence on plasma lipid levels. Subjects were instructed to avoid active weight loss, weight gain, or dietary changes for the study duration, and to avoid excessive alcohol consumption or physical activity before all clinic visits.

### 2.3. Sample Collection and Processing

Following an overnight fast (>10 h), venous blood was collected into ethylenediaminetetraacetic acid (EDTA) tubes on two consecutive days (36 mL on Day 1; 9 mL on Day 2) at baseline and post-intervention. Aliquots of plasma and erythrocytes isolated at 4 °C were stored at −80 °C for analysis of lipids and fatty acids. Plasma concentrations of TG and cholesterol were measured on each of the consecutive days at baseline and post-intervention, and the average of these measurements was used in statistical analyses. Fatty acid and lipoprotein measures were determined at baseline and post-intervention. Blood pressure was measured in triplicate at all clinic visits using an automatic blood pressure monitor. Body weight was recorded at baseline and post-intervention. The dietary macronutrient and micronutrient intakes of subjects were determined using the Victorian Anti-Cancer Council Food Frequency Questionnaire.

### 2.4. Clinical Chemistry Methods

Fasting plasma lipid levels (TG and total cholesterol (TC), HDL_2_, and HDL_3_ cholesterol) and isolated lipoprotein components (cholesterol, TG, phospholipids, and apolipoprotein B (apoB) were measured using an autoanalyser (Konelab 20XT) and commercially available kits, reagents, and standards from Thermo Electron, USA (cholesterol, TG), Kamiya Biomedical Company, USA (apoB), and Wako Pure Chemical Industries, Japan (phospholipids). Protein content within lipoproteins was determined using the Lowry method [23].

### 2.5. Lipoprotein Analysis

Isolation of lipoprotein fractions was conducted immediately by sequential ultracentrifugation of fresh plasma (10 mL) adjusted to appropriate densities with potassium bromide. VLDL, IDL, LDL, and HDL were isolated as the plasma fractions of densities <1.006 g/mL, 1.006–1.030 g/mL, 1.030–1.063 g/mL, and 1.063–1.21, respectively. This was achieved using ultracentrifugation and a 70.1Ti rotor at 39,000 rpm (139,439 g) at 10 °C for 16 h at a density of 1.006 g/mL, 18 h at a density of 1.030 g/mL, and 24 h at densities of 1.063 and 1.21 g/mL. The volumes of VLDL, IDL, LDL, HDL, and density > 1.21 g/mL samples were recorded to calculate recoveries, and aliquots were frozen at −80 °C for analysis of composition and particle size. The concentration of plasma HDL_2_ and HDL_3_ were determined using an established method [13]. Briefly, apoB containing lipoproteins were precipitated using heparin-manganese followed by enzymatic measurement of the remaining cholesterol. A second precipitation procedure using dextran sulfate was performed to determine the HDL_3_-C concentration. The concentration of HDL_2_ was calculated as the difference between the measured HDL-C and HDL_3_-C concentrations.

### 2.6. Lipoprotein Particle Size Analysis

Non-denaturing polyacrylamide gradient gels (Alamo Gels, USA) were used for the separation of IDL and LDL (2–16% gradient) and HDL subclasses (4–30% gradient) within isolated samples. Aliquots of IDL (31.5 nm), LDL_1_ (23.6 nm), and LDL_3_ (20 nm), for which particle sizes were previously determined using electron microscopy [24], were also run on the 2–16% gels to generate a standard curve for the determination of LDL particle size. Standards run on the 4–30% gradient gels included latex beads (38 nm), thyroglobulin (17.1 nm) ferritin, (12.2 nm), lactate dehydrogenase (8.16 nm), and albumin (7.1 nm). Following electrophoresis, gels were stained, scanned, and analysed using Image J version 1.43 u software (National Institutes of Health, Bethesda, MD, USA). Peak particle diameter was quantified for LDL samples. HDL particle diameter was obtained from a logarithmic standard curve of the diameter of standards against their positions on the scanned gel. HDL sub-classes were defined as HDL_2b_ (9.9–12.0 nm), HDL_2a_ (8.8–9.9 nm), HDL_3a_ (8.2–8.8 nm), HDL_3b_ (7.8–8.2 nm), or HDL_3c_ (7.0–7.8 nm) [25]. For these subclasses, the relative distribution of cholesterol under each peak was calculated as a percentage of the total area for all HDL subclasses.

### 2.7. Fatty Acid Analysis

The fatty acid profiles of erythrocytes were determined using standard methods reported previously [26]. Briefly, erythrocyte aliquots (400 μL) were thawed and re-suspended in a TRIS buffer (10 mM Bis Tris, 2 mM EDTA Na_2_, pH 7.2) at room temperature for 30 min. The samples were then spun in an ultracentrifuge at 315,000 g for 30 min at 4 °C (Beckman L-80 OPTIMA, Beckman Coulter, Burea, CA, USA) to pellet erythrocyte membranes. Upon removal of the supernatant, the erythrocyte membrane pellet was re-suspended in 200 μL of distilled water. A fixed volume (150 μL) of the erythrocyte membrane suspension was used for direct transesterification of fatty acids [27] using heneicosanoic acid as the internal standard. Fatty acid methyl esters (FAMEs) were analysed by injecting 1 μL of each sample in a gas chromatograph (GC 17A Shimadzu, Shimadzu Corp., Columbia, MD, USA) equipped with an autoinjector, 30 m FAME capillary column (0.25 mm internal diameter, Varian, Palo Alto, CA, USA), and flame ionization detector. Hydrogen was used as the carrier gas. Fatty acid peaks were identified by comparison to known mixed standards (Nu-Chek Prep, Waterville, MN, USA; Sigma Aldrich, Castle Hill, NSW, Australia), and quantified using Shimadzu software (Class-VP 7.2.1 SP1, Kyoto, Japan).

### 2.8. Statistical Analysis

Power calculations were based on the analysis of covariance (ANCOVA) method, as it accounts for baseline differences while avoiding regression to the mean [28] and has the greatest power for detecting treatment effects in randomised controlled trial data [28,29,30,31]. A sample size formula for analysis of covariance in randomized controlled trials was used [32], and the total number of participants required per group was 14. The Shapiro-Wilk test was used to assess whether each variable fit a normal distribution. All plasma lipid, lipoprotein, and dietary variables were found to be non-normal and were subsequently transformed using the log_10_ algorithm prior to statistical analyses.

Baseline differences between dose groups for all parameters were examined using one-way ANOVA. Comparisons between groups were based on ANCOVA standard least squares models with Tukey honest significant difference (HSD) analysis. *n*-3 LCPUFA dose and the baseline value for each parameter were used as the two covariates in ANCOVA, with the post-intervention value as the dependent variable. Percentage reductions in parameters were calculated on means of log-transformed data. For each parameter, a trend test was performed using the *n*-3 LCPUFA dose (0, 0.35, 0.7, or 1.0 g/day) as a continuous variable in a multiple regression model (standard least squares), the baseline value as a covariate, and the post-intervention value as the dependent variable. This multiple regression model was used to determine the statistical significance of dose (or baseline level) as a predictor of the post-intervention measure, as well as the combined predictive capacity of the two variables. Linear regression was used to determine the individual predictive capacity of each variable. Statistical analyses were conducted using JMP 5.1 statistical software (SAS, Cary, NC, USA).

## 3. Results

### 3.1. Subject Characteristics and Dietary Intake

Of the 64 eligible women who commenced the trial, 56 completed. Drop-outs were due to time-constraints or unexpected travel (*n* = 6), surgery (*n* = 1), and an adverse reaction (mouth ulceration) that may have been related to capsule consumption (*n* = 1). Three subjects who completed the trial were subsequently excluded from analysis (Figure 1). Fifty-three subjects were included in the final analysis of blood pressure and all lipid and lipoprotein compositions, and 29 were included in analysis of LDL and HDL particle size. Subject characteristics and nutrient intakes did not significantly differ between groups (Table 1).

### 3.2. Blood Pressure and Fatty Acids

The study population presented with normal baseline blood pressure, with a trend towards lower systolic (*p* = 0.07) but not diastolic (*p* = 0.58) blood pressure in the 1 g/day group (Table 1). After fish oil supplementation, both systolic (*p* = 0.16) and diastolic (*p* = 0.91) blood pressures were unaffected by *n*-3 LCPUFA dose. 

**Figure 2:**
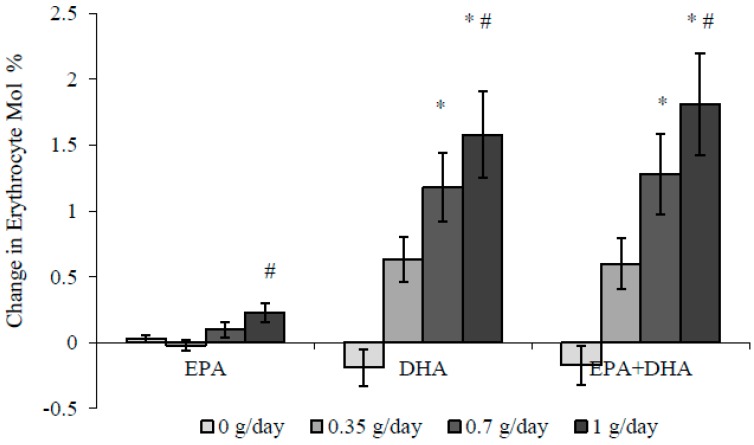
Changes in erythrocyte *n*-3 LCPUFA levels (mean ± SEM) following supplementation with 0–1 g/day *n*-3 LCPUFA from HiDHA™ tuna oil. * Significantly greater than 0 g/day using analysis of covariance (ANCOVA) (*p* < 0.05), # Significantly greater than 0.35 g/day using ANCOVA (*p* < 0.05). 0 g/day group *n* = 12; 0.35 g/day group *n* = 15; 0.7 g/day group *n* = 13; 1.0 g/day group *n* = 13. DHA: docosahexaenoic acid, EPA: eicosapentaenoic acid.

Erythrocyte EPA and DHA levels increased in a dose-dependent manner (Figure 2), resulting in a 15% increase in the Omega-3 Index (erythrocyte EPA + DHA as mol % of total fatty acids) after 0.35 g/day, and increases of 27% and 39% after 0.7 g/day and 1 g/day *n*-3 LCPUFA, respectively.

### 3.3. Effect of Dose–Response n-3 LCPUFA Supplementation on Plasma Lipids

**Figure 3:**
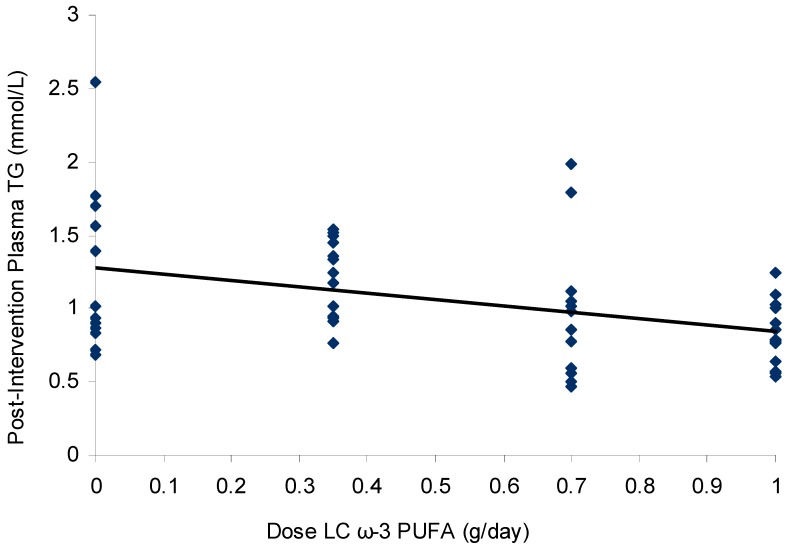
Scatterplot (with linear regression line) of plasma triglyceride levels (mmol/L) following supplementation with 0, 0.35, 0.7, and 1 g/day *n*-3 LCPUFA from HiDHA™ tuna oil (*n* = 53). *R*^2^ = 0.16, *p* = 0.003 in the per-protocol analysis. 0 g/day group *n* = 12; 0.35 g/day group *n* = 15; 0.7 g/day group *n* = 13; 1.0 g/day group *n* = 13.

The average baseline fasting TG level of the study cohort was 1.23 ± 0.47 mmol/L (mean ± standard deviation (SD)), with no difference between dose groups (*p* = 0.56). Individual TG levels ranged from 0.53–2.42 mmol/L, despite excluding fasting plasma TG levels of >1.0 mmol/L in the screening phase. BMI was a weak but significant predictor of baseline plasma TG levels (*R*^2^ = 0.09, *p* = 0.03). Following 8 weeks of supplementation with 1 g/day *n*-3 LCPUFA, plasma TG levels significantly lowered compared to placebo (Table 2). This change amounted to a 23% reduction from baseline. There was no difference between groups in baseline plasma TC levels (*p* = 0.98), and no dose–response effect following *n*-3 LCPUFA supplementation (*p* = 0.64). In an intention-to-treat analysis, plasma TG levels lowered according to increasing dose (*R*^2^ = 0.09, *p* = 0.04) within the supplemental range of 0–1 g/day *n*-3 LCPUFA. The dose effect increased upon a per-protocol analysis (*R*^2^ = 0.16, *p* = 0.003) (Figure 3). When BMI was included in the model in addition to dose and baseline TG levels, the variability in post-intervention TG levels explained by the model increased slightly, from *R*^2^ = 0.45 to *R*^2^ = 0.48.

### 3.4. Lipoprotein Composition and Particle Size

There was no difference between dose groups in baseline levels of any lipoprotein particle components (triglyceride, cholesterol, phospholipid, protein, or apoB). The composition of isolated VLDL particles at baseline and after supplementation with *n*-3 LCPUFA is presented in Table 3. There were no significant differences between groups at baseline for the concentrations or percentage contribution of VLDL particle components. Post-intervention VLDL-TG levels in the group supplemented with 1 g/day *n*-3 LCPUFA were significantly lower (32%) than the 0.35 g/day group; however, they were not significantly lower than placebo due to the apparent VLDL-TG reduction in this group (Table 3).

**Figure 4:**
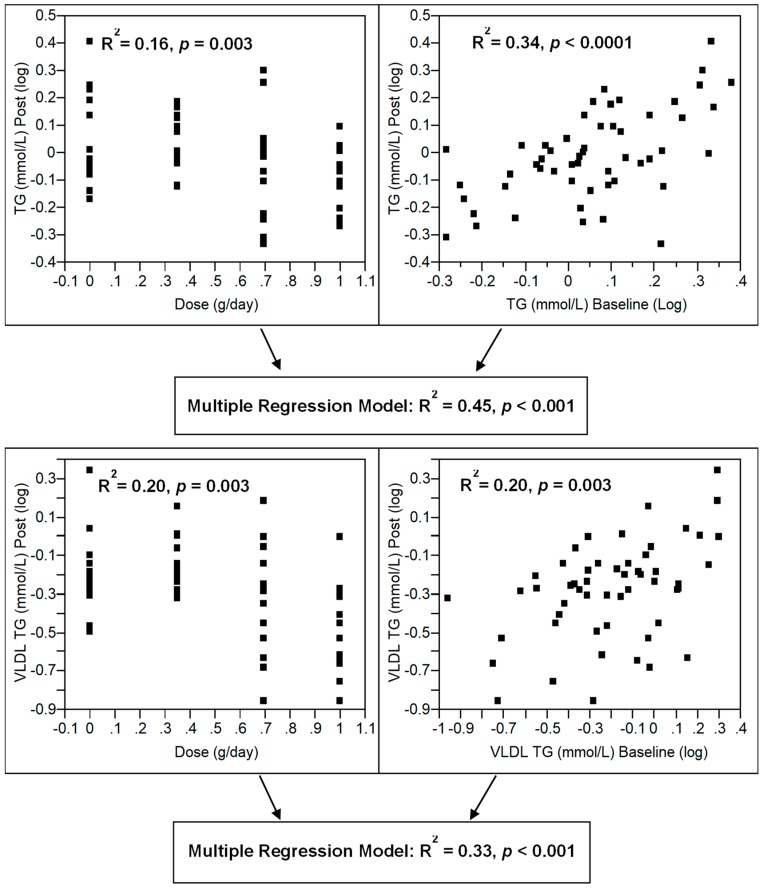
Multiple regression models for the combined effects of *n*-3 LCPUFA dose and baseline levels of plasma and very-low-density lipoprotein (VLDL) triglyceride levels in pre-menopausal women supplementation with 0, 0.35, 0.7, and 1 g/day *n*-3 LCPUFA from HiDHA™ tuna oil (*n* = 53). 0 g/day group *n* = 12; 0.35 g/day group *n* = 15; 0.7 g/day group *n* = 13; 1.0 g/day group *n* = 13.

Lipid components of VLDL reduced in a dose-dependent manner despite no change in VLDL apoB levels (Table 3), suggesting a reduction in VLDL particle size. As demonstrated by compositional analysis of all TG-carrying lipoproteins (Table 3 and Table 4), the reduction in plasma TG was entirely due to a reduction in VLDL-TG. Baseline levels were also a predictor of post-intervention plasma TG and VLDL-TG (Figure 4). When combined with dose in a multiple regression model, plasma TG and VLDL-TG explained 45% and 33% of the variability in post-intervention levels, respectively (Figure 4).

IDL, LDL, and HDL particle compositions were unaffected by *n*-3 LCPUFA supplementation (Table 4). The significant difference between HDL3-C levels in the placebo group and the 0.35 and 0.7 g/day groups was driven by an unexplained increase in the placebo group rather than a reduction in the treatment groups.

**Figure 5:**
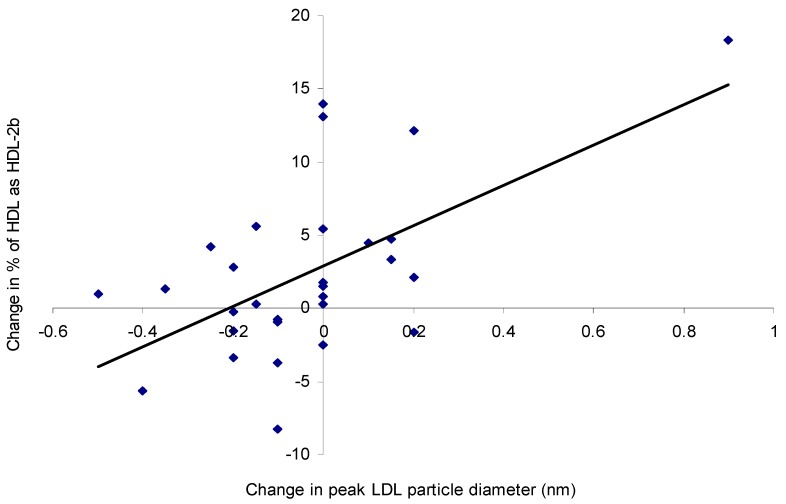
Scatterplot showing the relationship between the change in LDL particle diameter (nm) and the change in the proportion of HDL_2b_ following supplementation with 0, 0.35, 0.7, and 1 g/day *n*-3 LCPUFA from HiDHA™ tuna oil (*n* = 29). *R*^2^ = 0.34, *p* = 0.0008. 0 g/day group *n* = 12; 0.35 g/day group *n* = 15; 0.7 g/day group *n* = 13; 1.0 g/day group *n* = 13.

The dominant LDL particle type was LDL2, with small dense LDL3 particles present in very few subjects and no particle size change upon *n*-3 LCPUFA supplementation (Table 5). There was a very weak dose-dependent increase in the proportion of HDL2b particles but not in HDL2a or HDL3a (Table 5). The change in plasma TG levels was a predictor of the change in the proportion of HDL2b particles (*R*^2^ = 0.15, *p* = 0.04). Similarly, the change in LDL peak particle size was a predictor of the change in the proportion of HDL2b (*R*^2^ = 0.34, *p* = 0.0008) (Figure 5), HDL3b (*R*^2^ = 0.20, *p* = 0.01), and HDL2a (*R*^2^ = 0.28, *p* = 0.003) particles.

## 4. Discussion

This was the first double-blind, randomized, placebo-controlled study that assessed the effect of *n*-3 LCPUFA supplementation on plasma lipids and lipoproteins in pre-menopausal women where the confounding effects of the menstrual cycle were strictly controlled. The results revealed that a low dose of *n*-3 LCPUFA is effective for lowering plasma and VLDL TG levels in pre-menopausal women. Supplementation with 1 g/day of *n*-3 LCPUFA from HiDHA™ tuna oil reduced fasting plasma TG by 23% compared to placebo. Supplementation with a wide range of DHA doses (0.94–5.7 g/day) in healthy and hypertriglyceridemic populations have produced remarkably similar reductions in plasma TG (20–32%) [5,6,7,9,10,11,14,15,33,34,35,36,37], suggesting there could be a plateau effect at *n*-3 LCPUFA doses greater than 1 g/day. The TG-reduction between 0–1 g/day appeared to be dose-dependent in a linear fashion, despite the lower doses not significantly lowering TG compared to placebo. These results are in line with previous reports in mixed gender populations of a lack of TG-lowering with approximately 0.7 g/day of DHA [9,16] or *n*-3 LCPUFA [38]. Baseline TG levels influenced the TG-lowering effect of the supplement. This was in agreement with previous reports that individuals with higher baseline TG levels respond more strongly to supplementation [2]. Substantial inter-individual variability in plasma TG response was recorded, as noted by others [39,40]. Supplementation with 1 g/day *n*-3 LCPUFA produced TG changes ranging from a 49% reduction to a 38% increase from baseline. All other doses resulted in similarly broad ranges of response. This variability likely contributed to the relatively weak effect of dose on plasma TG. Hence, research should be conducted in larger study populations to further elucidate dose–response relationships at low *n*-3 LCPUFA intakes. 

While the protocol strictly controlled for daily variation in TG levels, it is possible there were uncontrolled factors that were not accounted for. Erythrocyte fatty acid analysis confirmed the self-report measures that compliance was good, thus the observed variability in TG response is unlikely due to lack of compliance with capsule consumption. However, similarly to as noted by others [41], no linear relationship between the change in erythrocyte *n*-3 LCPUFA levels and the change in plasma TG was detected. Polymorphisms in the genes for fatty acid translocase (FAT)/CD36 [42], PPAR-α [43], and apolipoproteins [44,45] are known to contribute to variability in TG response to *n*-3 LCPUFA supplementation. Apolipoprotein E (apoE) genotype in particular has been studied [46], however, it appears to have little to no influence on the effect of fish oil in women [47] or on fasting (as opposed to post-prandial) plasma TG [39,48]. As dietary nutrient intakes were measured only once rather than at baseline and post-intervention, it is possible that subjects altered their diet during the study period. Some subjects may also have failed to comply with the fasting protocol or instructions to avoid alcohol consumption and intense exercise prior to clinic visits. 

The mechanisms behind plasma lipid responses to *n*-3 LCPUFA consumption are not fully understood, and may differ for EPA and DHA. However, TG reductions appear to be driven by reduced hepatic VLDL production. SREBP-1c, the hepatic gene transcription factor that regulates endogenous triglyceride production, is markedly inhibited by *n*-3 LCPUFA [49,50]. Coupled with increased fatty acid oxidation by activation of PPAR-α [51], these actions leave less substrate for TG synthesis and packaging into VLDL. Therefore, as shown in this study, VLDL particles tend to be smaller and less TG-rich. Moderate-high doses of DHA (2–3 g/day) reduce VLDL particle size [7,34]. Low dose *n*-3 LCPUFA (0.8 g/day) has also been reported to reduce VLDL lipid, protein, and apoB levels, suggesting a reduction in particle size and number [52]. However, the latter study was limited by the lack of a placebo-control, small sample size, and short duration. In the present study, not only was the plasma TG reduction reflected in a dose-dependent reduction of VLDL-TG levels, their changes were highly related (*R*^2^ = 0.53, *p* < 0.0001). In contrast, VLDL protein and apoB levels were unchanged, suggesting that the number of particles secreted by the liver was unaffected. The dose-dependent increase in VLDL-TG to protein ratio, and changes in their percentages within VLDL particles, also indicates a reduction in core lipid components but no change in surface constituents. Furthermore, IDL composition did not change as a result of *n*-3 LCPUFA supplementation; hence, taken together, these results suggest that smaller VLDL particles were secreted as a result of low-dose HiDHA™ tuna oil supplementation, thereby reducing plasma TG levels, whilst there was no effect on IDL particles.

A reduction in TG substrate for cholesterol ester transfer protein (CETP) limits the exchange of VLDL TG for cholesterol from HDL and/or LDL, which could lead to the maintenance of larger, more buoyant LDL and HDL particles. However, in this study HiDHA™ tuna oil had no effect on LDL composition or particle size in pre-menopausal women. This was not surprising, given that LDL-C was normal at baseline and small dense LDL particles were not prominent. While other studies reported no change in LDL-C after supplementation with low doses of *n*-3 LCPUFA [38,53], DHA intakes as low as 0.7–1.5 g/day have been shown to increase LDL-C [5,6,9,14,16] and LDL particle size [6] or cholesterol:apoB ratio [16]. As with plasma TG changes, apoE genotypes appear to alter LDL response to *n*-3 LCPUFA supplementation [39], perhaps with DHA but not EPA [48]. However, Caslake et al. [47] found no genotype-dependent effect with 0.7 or 1.8 g/day, thus suggesting that this effect may also be dose-dependent. In this study, a very minor dose-dependent change in cholesterol distribution from the smaller, denser HDL_3b_ and HDL_3c_ particles to the larger, more buoyant HDL_2b_ particles was observed. More importantly, a positive correlation existed between the increases in the proportion of HDL_2b_ particles and LDL particle size, suggesting a reduction in CETP-mediated exchange of triglyceride and cholesterol between lipoproteins in some individuals. The lack of an effect on total HDL-C was in agreement with the literature, whether low to moderate [9,54] or even high [40] doses of *n*-3 LCPUFA were administered. Modest increases in the proportion of HDL_2_, and reductions in HDL_3_, have been reported with as little as 0.7 g/day *n*-3 LCPUFA [47]. However, males responded twice as strongly as females [47]; therefore, an effect in premenopausal women was unlikely.

Low dose HiDHA™ tuna oil had no effect on blood pressure in this study population. While high doses (>3 g/day *n*-3 LCPUFA) are generally required to reduce blood pressure in hypertensive patients [21,55], reductions in systolic blood pressure have been demonstrated following supplementation with low doses (1 g/day *n*-3 LCPUFA) of both seal oil and HiDHA™ tuna oil [22]. However, the average blood pressure in the aforementioned study was 130/75 mmHg [22]; it is likely that the comparatively lower baseline blood pressure in this cohort of pre-menopausal women did not reach the threshold at which *n*-3 LCPUFA would provide a benefit.

A significant strength of this study was the strict control of confounding effects of the menstrual cycle on plasma lipids. Indeed, this is the first study to do so and demonstrate the plasma TG-lowering effect of *n*-3 LCPUFA in pre-menopausal women. Despite preliminary screening to exclude women with low plasma TG levels, 16 subjects exhibited baseline plasma TG levels lower than the minimum for entry to the study. For these participants, there remained little room for further reductions in TG through HiDHA™ tuna oil supplementation. However, even with this limitation, the percentage reduction in TG observed in this study after supplementation with 1.0 g/day was similar to reductions in hypertriglyceridemic subjects and with substantially greater doses of *n*-3 LCPUFA.

## 5. Conclusions

Supplementation with 1 g/day HiDHA™ tuna oil is sufficient to lower plasma TG by approximately 20% in pre-menopausal women, and the benefit is of a similar degree to that observed in men and post-menopausal women. The reduction in plasma TG was not accompanied by an increase in LDL cholesterol or particle size. There may also be some benefit of HiDHA™ tuna oil to HDL particle size in premenopausal women. Consumption in this dose range may be of particular significance in maintaining cardiovascular health in pre-menopausal women, given the greater contribution of TG levels to their CVD risk.

## Figures and Tables

**Table 1:** Characteristics (values mean ± standard error of the mean (SEM) or median (25th percentile, 75th percentile); *n* = 53) and daily dietary energy (kJ/day) and macronutrient intakes (g/day) (values median (25th percentile, 75th percentile); *n* = 45) of subjects in double-blind placebo-controlled trial at baseline.

	0 g/day (*n* = 12)	0.35 g/day (*n* = 15)	0.7 g/day (*n* = 13)	1 g/day (*n* = 13)	*p* Value
*N*	12	15	13	13	
OC/Non-OC	6/6	7/8	5/8	7/6	
Age (years)	28 ± 2	27 ± 2	24 ± 1	28 ± 2	0.51
BMI (kg/m^2^)	23 ± 1	26 ± 1	26 ± 2	24 ± 1	0.57
SBP (mm Hg)	116 (108, 120)	117 (107, 121)	115 (110, 125)	110 (105, 112)	0.07
DBP (mm Hg)	69 (65, 75)	70 (68, 80)	70 (65, 82)	69 (66, 72)	0.58
TC (mmol/L)	4.60 (4.02, 5.42)	4.61 (4.13, 4.88)	4.30 (4.14, 4.95)	4.60 (3.90, 5.22)	0.98
Energy (kJ)	6525 (5696, 7475)	5871 (5005, 6888)	6642 (5460, 8081)	5118 (4115, 6365)	0.24
Total Fat (g)	50 (47, 71)	58 (48, 70)	60 (49, 75)	43 (31, 61)	0.44
SFA (g)	24 (20, 29)	23 (18, 28)	24 (20, 30)	18 (12, 25)	0.50
PUFA (g)	7.1 (5.6, 9.0)	8.2 (6.4, 11)	8.6 (6.4, 12)	5.6 (3.6, 8.6)	0.34
MUFA (g)	21 (16, 26)	22 (18, 26)	21 (17, 27)	16 (11, 22)	0.44
Protein (g)	81 (69, 94)	72 (62, 84)	81 (67, 99)	70 (60, 82)	0.44
CHO (g)	179 (162, 197)	148 (126, 174)	177 (142, 221)	136 (113, 164)	0.09

Abbreviations: OC/Non-OC: Oral Contraceptive Use/Non-Use; BMI: Body Mass Index; SBP: Systolic Blood Pressure; DBP: Diastolic Blood Pressure; TC: Total Cholesterol; SFA: Saturated fatty acids; PUFA: polyunsaturated fatty acids; MUFA: monounsaturated fatty acids; CHO: Carbohydrate.

**Table 2:** Plasma triglyceride and cholesterol levels (mmol/L) before and after supplementation with 0–1 g/day *n*-3 LCPUFA from HiDHA™ tuna oil (median (25th percentile, 75th percentile); *n* = 53).

*n*-3 LCPUFA (g/day)	TG (mmol/L) 0 weeks	TG (mmol/L) 8 weeks	TC (mmol/L) 0 weeks	TC (mmol/L) 8 weeks
0.00 (*n* = 12)	1.18 (0.77, 1.57)	0.98 (0.84, 1.66)	4.60 (4.02, 5.42)	4.68 (3.89, 5.05)
0.35 (*n* = 15)	1.27 (1.07, 1.69)	1.18 (0.91, 1.45)	4.61 (4.13, 4.88)	4.43 (4.04, 4.75)
0.70 (*n* = 13)	1.07 (0.91, 1.86)	0.98 (0.58, 1.08)	4.30 (4.14, 4.95)	4.42 (4.07, 4.87)
1.00 (*n* = 13)	1.09 (0.78, 1.27)	0.79 * (0.60, 1.02)	4.60 (3.90, 5.22)	4.44 (3.85, 5.41)

* Significantly greater than 0 g/day using ANCOVA (*p* < 0.05). Abbreviations: *n*-3 LCPUFA: *n*-3 Long Chain Polyunsaturated Fatty Acids; TG: Triglycerides; TC: Total Cholesterol.

**Table 3:** Changes in VLDL particle composition following supplementation with 0–1 g/day *n*-3 LCPUFA from HiDHA™ tuna oil (values median (25th percentile, 75th percentile); *n* = 53).

VLDL	0 g/day (*n* = 12) 0 weeks	0 g/day (*n* = 12) 8 weeks	0.35 g/day (*n* = 15) 0 weeks	0.35 g/day (*n* = 15) 8 weeks	0.7 g/day (*n* = 13) 0 weeks	0.7 g/day (*n* = 13) 8 weeks	1 g/day (*n* = 13) 0 weeks	1 g/day (*n* = 13) 8 weeks	Dose Effect (*R*^2^, *p* Value)
TG (mmol/L)	0.81 (0.51, 1.23)	0.60 (0.50, 0.77)	0.72 (0.49, 0.94)	0.66 (0.58, 0.85)	0.96 (0.31, 1.37)	0.51 (0.26, 0.80)	0.50 (0.35, 0.77)	0.35 ^#^ (0.22, 0.52)	*R*^2^ = 0.20, *p* = 0.003
Chol (mmol/L)	0.36 (0.19, 0.47)	0.19 (0.16, 0.48)	0.31 (0.17, 0.40)	0.24 (0.18, 0.35)	0.30 (0.14, 0.63)	0.19 (0.11, 0.29)	0.21 (0.15, 0.30)	0.16 (0.09, 0.24)	*R*^2^ = 0.14, *p* = 0.02
PL (mg/dL)	21 (13, 32)	15 (12, 29)	19 (12, 22)	15 (14, 19)	21 (9, 41)	14 (7, 19)	16 (10, 20)	11 (6, 17)	*R*^2^ = 0.17, *p* = 0.003
PR (mg/dL)	14 (10, 17)	12 (10, 15)	18 (12, 21)	12 (9, 17)	15 (9, 22)	11 (9, 14)	10 (9, 15)	11 (8, 13)	*p* = 0.33
TG/PR	5.4 (3.1, 7.5)	5.0 (3.4, 6.6)	3.9 (3.3, 5.5)	6.0 (3.5, 6.7)	4.0 (2.4, 8.1)	3.9 (2.6, 5.3)	4.1 (2.4, 5.4)	2.4 ^#^ (2.2, 4.2)	*R*^2^ = 0.17, *p* = 0.003
apoB (mmol/L)	6.3 (3.3, 9.0)	5.2 (3.5, 6.2)	7.7 (6.5, 9.6)	5.7 (4.8, 6.5)	5.5 (4.2, 7.2)	4.3 (3.2, 6.6)	5.0 (4.0, 8.0)	4.7 (2.5, 5.5)	*p* = 0.49
TG %	58 (55, 61)	57 (53, 63)	57 (49, 60)	58 (56, 62)	57 (43, 60)	55 (50, 59)	51 (49, 58)	50 ^#^ (48, 55)	*R*^2^ = 0.13, *p* = 0.02
Chol %	10 (10, 12)	9 (8, 11)	9 (8, 11)	10 (8, 11)	10 (8, 13)	9 (8, 11)	10 (9, 11)	9 (8, 11)	*p* = 0.97
PL %	17 (16, 18)	16 (16, 19)	17 (15, 17)	16 (15, 17)	16 (16, 19)	17 (15, 18)	18 (16, 19)	18 (16, 19)	*p* = 0.82
PR %	11 (8, 18)	12 (9, 17)	14 (11, 17)	10 (9, 16)	15 (7, 19)	15 (11, 21)	14(11, 21)	21 ^#^ (13, 23)	*R*^2^ = 0.16, *p* = 0.002

Differences between dose groups compared using log-transformed data with ANCOVA followed by post hoc Tukey honest significant difference (HSD) tests. Abbreviations: VLDL: very low density lipoprotein, TG: triglyceride, Chol: cholesterol, PL: phospholipids; PR: protein, apoB: apolipoprotein B, ^#^ Significantly lower than 0.35 g/day using ANCOVA (*p* < 0.05).

**Table 4:** Changes in lipoprotein composition following supplementation with 0–1 g/day *n*-3 LCPUFA from HiDHA™ tuna oil (values median (25th percentile, 75th percentile); *n* = 53).

	0 g/day (*n* = 12) 0 weeks	0 g/day (*n* = 12) 8 weeks	0.35 g/day (*n* = 15) 0 weeks	0.35 g/day (*n* = 15) 8 weeks	0.7 g/day (*n* = 13) 0 weeks	0.7 g/day (*n* = 13) 8 weeks	1 g/day (*n* = 13) 0 weeks	1 g/day (*n* = 13) 8 weeks	Dose Effect (*R*^2^, *p* Value)
IDL-C (mmol/L)	0.33 (0.26, 0.40)	0.37 (0.28, 0.44)	0.37 (0.29, 0.53)	0.35 (0.27, 0.50)	0.37 (0.22, 0.46)	0.30 (0.20, 0.40)	0.36 (0.24, 0.49)	0.41 (0.20, 0.50)	*p* = 0.17
IDL-TG (mmol/L)	0.15 (0.10, 0.17)	0.14 (0.11, 0.16)	0.15 (0.13, 0.19)	0.15 (0.12, 0.17)	0.11 (0.09, 0.14)	0.11 (0.08, 0.12)	0.14 (0.11, 0.17)	0.12 (0.10, 0.18)	*p* = 0.37
IDL-apoB (mmol/L)	10 (7.5, 11)	11 (9.4, 13)	11 (9.7, 12)	11 (7.5, 13)	8.7 (6.7, 11)	7.8 (6.2, 13)	11 (7.5, 15)	14 (7.9, 17)	*p* = 0.65
LDL-C (mmol/L)	2.6 (2.0, 2.8)	2.0 (1.7, 3.0)	2.5 (1.7, 2.8)	2.4 (1.9, 2.8)	2.0 (1.8, 2.3)	2.2 (2.0, 2.5)	2.2 (1.8, 3.1)	2.3 (1.7, 2.8)	*p* = 0.75
LDL-TG (mmol/L)	0.17 (0.11, 0.21)	0.16 (0.13, 0.23)	0.18 (0.15, 0.20)	0.17 (0.15, 0.20)	0.14 (0.11, 0.18)	0.13 (0.11, 0.16)	0.17 (0.14, 0.22)	0.17 (0.14, 0.21)	*p* = 0.64
LDL-apoB (mmol/L)	113 (70.3, 121)	99.5 (61.1, 118)	94.6 (64.3, 118)	115 (75.6, 123)	68.0 (43.9, 98.2)	77.1 (57.4, 120)	107 (77.7, 146)	102 (84.9, 135)	*p* = 0.92
HDL-C (mmol/L)	1.2 (0.94, 1.4)	1.3 (0.98, 1.6)	1.1 (0.89, 1.2)	1.2 (0.96, 1.4)	1.2 (0.94, 1.6)	1.2 (1.0, 1.5)	1.2 (1.0, 1.3)	1.2 (0.99, 1.5)	*p* = 0.26
HDL_2_-C (mmol/L)	0.59 (0.41, 0.62)	0.59 (0.45, 0.71)	0.49 (0.35, 0.55)	0.56 (0.47, 0.71)	0.61 (0.38, 0.77)	0.65 (0.40, 0.79)	0.54 (0.40, 0.75)	0.66 (0.44, 0.77)	*p* = 0.98
HDL_3_-C (mmol/L)	0.65 (0.51, 0.79)	0.73 (0.52, 0.83)	0.62 (0.54, 0.68)	0.61 * (0.50, 0.68)	0.63 (0.57, 0.77)	0.60 * (0.55, 0.71)	0.61 (0.52, 0.76)	0.62 (0.49, 0.78)	*R*^2^ = 0.01, *p* = 0.04

Abbreviations: IDL: intermediate density lipoprotein, LDL: low density lipoprotein, HDL: high density lipoprotein, TG: triglyceride, C: cholesterol, apoB: apolipoprotein B; * Significantly lower than 0 g/day using ANCOVA (*p* < 0.05).

**Table 5:** Lipoprotein particle size following supplementation with 0–1 g/day *n*-3 LCPUFA from HiDHA™ tuna oil (values median (25th percentile, 75th percentile); *n* = 29).

	0 g/day (*n* = 12) 0 weeks	0 g/day (*n* = 12) 8 weeks	0.35 g/day (*n* = 15) 0 weeks	0.35 g/day (*n* = 15) 8 weeks	0.7 g/day (*n* = 13) 0 weeks	0.7 g/day (*n* = 13) 8 weeks	1 g/day (*n* = 13) 0 weeks	1 g/day (*n* = 13) 8 weeks	Dose Effect (*R*^2^, *p* Value)
LDL Radius (nm)	10.9 (10.8, 11.0)	10.9 (10.7, 10.9)	11.0 (10.4, 11.1)	10.9 (10.4, 11.1)	10.9 (10.6, 11.1)	10.7 (10.5, 11.0)	10.9 (10.7, 11.0)	10.8 (10.8, 11.3)	*p* = 0.15
% HDL_2b_	37 (32, 43)	33 (31, 44)	35 (16, 38)	35 (23, 38)	41 (28, 49)	46 (38, 50)	32 (28, 47)	42 (27, 52)	*R*^2^ = 0.05, *p* = 0.04
% HDL_2a_	30 (27, 33)	28 (26, 37)	24 (22, 31)	25 (22, 31)	26 (21, 35)	26 (21, 32)	31 (22, 33)	31 (24, 33)	*p* = 0.22
% HDL_3a_	19 (15, 23)	20 (15, 23)	20 (18, 29)	19 (17, 27)	19 (15, 25)	18 (14, 22)	18 (16, 20)	17 (13, 19)	*p* = 0.24
% HDL_3b_	12 (7, 14)	11 (7, 16)	13 (10, 20)	13 (10, 18)	11 (7, 14)	9 (2, 12)	17 (11, 18)	9 (8, 19)	*R*^2^ = 0.02, *p* = 0.07
% HDL_3c_	0.8 (0.0, 3.3)	0.3 (0.0, 3.9)	6.0 (0.6, 11.5)	6.1 (0.0, 12.5)	1.2 (0.0, 8.2)	1.9 (0.0, 8.2)	1.2 (0.6, 3.0)	0.6 (0.0, 2.1)	*p* = 0.10

Abbreviations: LDL: low density lipoprotein; HDL: high density lipoprotein.
